# Structure, Optical Properties and Physicochemical Features of LiNbO_3_:Mg,B Crystals Grown in a Single Technological Cycle: An Optical Material for Converting Laser Radiation

**DOI:** 10.3390/ma16134541

**Published:** 2023-06-23

**Authors:** Mikhail Palatnikov, Olga Makarova, Alexandra Kadetova, Nikolay Sidorov, Natalya Teplyakova, Irina Biryukova, Olga Tokko

**Affiliations:** 1 Tananaev Institute of Chemistry—Subdivision of the Federal Research Centre, Kola Science Centre of the Russian Academy of Sciences (ICT RAS), 184209 Apatity, Russia; o.makarova@ksc.ru (O.M.); ttyc9@mail.ru (A.K.); n.sidorov@ksc.ru (N.S.); n.tepliakova@ksc.ru (N.T.); oksanakrav@mail.ru (I.B.); 2 Solid State Physics Department, Petrozavodsk State University, 185910 Petrozavodsk, Russia; solvak@yandex.ru

**Keywords:** single crystals, lithium niobate, doping, dopant distribution coefficient, defect structure, microstructure, optical microscopy and spectroscopy, laser conoscopy, photoinduced light scattering

## Abstract

Two series of LiNbO_3_:Mg:B crystals have been grown and studied. Two doping methods—have been used. The crystals—have been co-doped with Mg and a non-metallic dopant, B. The physicochemical features of the growth—have been considered for LiNbO_3_:Mg:B crystals obtained from a boron-doped melt. The charge—has been prepared using different technologies: homogeneous (HG) and solid-phase (SP) doping. The same two methods have been used to grow single-doped LiNbO_3_:Mg crystals. A control near-stoichiometric (NSLN) crystal—has been grown via the HTTSSG (high-temperature top-seeded solution growth) method from a congruent melt (Li/Nb ≈ 0.946) with 5.5 wt% K_2_O. The characteristics of the LiNbO_3_:Mg:B crystals—have been compared with those of the LiNbO_3_:Mg and NSLN crystals. Physicochemical and structural reasons have been established for the differences in the distribution coefficients of magnesium (K_D_) during the growth of the HG- and SP-doped LiNbO_3_:B:Mg and LiNbO_3_:Mg crystals. The optical characteristics of the LiNbO_3_:B:Mg crystals—have been studied via optical spectroscopy, laser conoscopy and photoinduced light scattering (PILS). The influence of boron on the microstructure, compositional and optical uniformities and optical damage resistance of the LiNbO_3_:Mg:B crystals—has been estimated. Optimal technological approaches to growing optically uniform LiNbO_3_:B:Mg crystals have been determined. LiNbO_3_:Mg:B crystals have been shown to have a significant advantage over the commercially used LiNbO_3_:Mg crystals since large LiNbO_3_:Mg:B crystals can be grown without stripes. Such stripes usually appear perpendicular to the growth axis. In addition, the photorefractive effect is suppressed in LiNbO_3_:Mg:B crystals at lower magnesium concentrations ([Mg] ≈ 2.5 mol%) than in LiNbO_3_:Mg ([Mg] ≈ 5.5 mol%).

## 1. Introduction

The lithium niobate crystal (LN, LiNbO_3_) is one of the most practically important nonlinear optical materials [1,2,3]. LN is a phase of variable composition with a wide region of homogeneity on its phase diagram (~44–50.5 mol% Li_2_O) [1,3]. According to works [4,5,6,7], the unit cell of the ferroelectric phase of LN is characterized by a space group of symmetry C^6^_3V_ (R3c).

It is customary to change the physical characteristics of LN crystals by varying their stoichiometry (R = [Li]/[Nb]) and via doping with metal cations. The charges of the cations should be intermediate and between those of the Li^+^ and Nb^5+^. Doping with large (~2–7 mol%) concentrations of non-photorefractive metal dopants (Mg, Zn, Gd, In, Sc, etc.) usually leads to the formation of complex structural defects and compositional non-uniformity in the LN crystal [8].

Non-metallic cations have different chemical bonding mechanisms and other mechanisms of influence on the physicochemical characteristics of the crystal–melt system than those of metallic cationic dopants. Non-metallic cations are not able to enter the O_6_ oxygen octahedra in the structures of oxygen octahedral compounds (for example, LiNbO_3_). The oxides of reactive non-metallic elements (for example, boron) change the physicochemical characteristics and structure of the melt and change the structure and physical characteristics of the LN crystal [9,10,11]. The dopant oxides are used as a flux when growing LN crystals. The Curie temperature (T_C_) of LiNbO_3_:B crystals increases by ~40 K compared to a nominally pure congruent (CLN, R = Li/Nb = 0.946) crystal. The optical characteristics of the LiNbO_3_:B crystal change noticeably: the photoelectric fields (photovoltaic and diffusion) and the optical uniformity increase, and the photorefraction effect significantly decreases [9,10,11,12]. The concentration of boron in LiNbO_3_:B crystals is extremely low (~10^−5^–5 × 10^−4^ wt%) [9]. Boron almost does not incorporate into LiNbO_3_:B crystals.

Model calculations have been carried out on the basis of a fragment of a LiNbO_3_ crystal structure (cluster). The calculations have shown that B^3+^ cations, which have very small ionic radii (~0.15 Å), are able to localize not in octahedral O_6_ but in much smaller tetrahedral voids as part of the [BO_3_]^3−^ groups of the LiNbO_3_ crystal structure. The calculations have determined the possible positions of the B^3+^ cation in the structure of the LN crystal [9,13].

Raman spectroscopy data were previously obtained in LiNbO_3_:B crystals in the range of the vibrational frequencies of O_6_ oxygen octahedra (~550–900 cm^−1^); IR absorption spectroscopy data were obtained in the region of the stretching vibrations of OH^-^ groups (~3420–3550 cm^−1^). According to the data, MeO_6_ oxygen octahedral clusters are quite strongly distorted (Me are the main Li, Nb, and impurity metal cations) [9,12,14] in LN.

For the first time, a full-profile analysis of XRD patterns (the Rietveld method) showed that composition and structure of LiNbO_3_:B crystals are close to those of SLN crystals.In addition, for the first time, the increase in the chemical purity of LiNbO_3_:B crystals was experimentally confirmed: the melt in the Li_2_O–Nb_2_O_5_–B_2_O_3_ system is purified by the formation of strong boron-containing compounds with impurity metal cations. The compounds are thus removed from the crystallization process. This was substantiated by thermodynamic calculations in work [15].

Physicochemical characteristics were considered in [15]. Features of the crystallization of a boron-containing melt were considered on the basis of these characteristics. These features help to obtain compositionally and optically uniform LiNbO_3_:B crystals.

It is possible to grow a homogeneous LiNbO_3_:B crystal with suppressed photorefraction if up to 50% of the mass of the melt is crystallized. When heavily doped LiNbO_3_:Zn or LiNbO_3_:Mg crystals with suppressed photorefraction are grown, the melt fraction does not exceed 20–25% [16,17].

In general, a large number of articles have been devoted to LN crystals that were single-, double-, and even triple-doped with various metals in recent years [18,19,20,21,22,23]. Doping was carried out in order to reduce photorefractive sensitivity and create laser and holographic materials, etc. For example, the authors of [18] studied LN:Bi. In [19], triple-doped LiNbO_3_:Ho/Yb/Tm crystals were considered. In [20], triple-doped In:Ru:Fe:LiNbO_3_ crystals were studied. In [21], the properties of single-doped In:LiNbO_3_ crystals were revealed. The authors of [22] studied LN co-doped with Cu^2+^ and Ni^+^ in detail. Photorefraction was studied in [23] in vanadium, iron, and zirconium co-doped LN single crystals. The studies are varied and numerous, and we examine here only a few examples.

In recent years, a number of pioneering works have been devoted to a noticeable change in the fundamentally important optical characteristics of LN crystals when they are doped with tetravalent dopants [24,25,26,27,28,29,30]. The results of studying the increase in photorefraction in the UV region upon doping LN crystals with alkaline earth metals and its suppression upon doping with tetravalent dopants are particularly impressive. Doping with tetravalent dopants in much lower concentrations than with Mg significantly suppresses the photorefractive effect in the visible region.

Most recently, articles devoted to the study of double-doped LN crystals have been highlighted in which one of the doping metal components is magnesium [31,32,33,34,35,36,37,38,39,40,41,42,43,44,45,46,47]. The authors of [31] studied Mg-doped and Zn-doped crystals. Work [32] is dedicated to theoretical studies of pure and Mg^2+^-, Sc^3+^-, and Zr^4+^-doped LN. The authors of [33] grew and analyzed co-doped V:Mg:Ln and V:Fe:Zr:LN crystals. In [34], a crystallization electromotive force was studied in congruent and Mg-doped LN. The relationship between the melt and solid crystal was considered in [35], which introduced 4.7 mol% of MgO into LN with 50 mol% of Nb_2_O_5_. The authors of [36] co-doped Pr-doped LN with Mg ions: Pr:CLN (Pr = 1.06 mol%) and Pr:Mg:CLN (Pr = 1.09 mol%, Mg = 5.21 mol%). The authors of [37] studied the properties of treated, pure LN and LN:Mg crystals. The authors of [38] prepared pressed pellets of LiNbO_3_:Pr, LiNbO_3_:Mg,Pr, and LiTaO_3_:Pr. An interface electric field was applied during growth using the micro-pulling-down technique for congruent and Mg-doped LN crystals in [39]. The authors of [40] explored Mo and Mg co-doped LiNbO_3_. A series of thermoluminescence experiments was performed upon co-doped Mg^2+^:Pr^3+^:LiNbO_3_ crystals in [41]. In [42], 2.0, 4.0, and 6.0 mol% of Mg-doped LN single crystals were grown via the Czochralski method. Fano-resonant Si nanoparticles were introduced into a co-doped Mg:Er:LiNbO_3_ single crystal in [43]. The absorption of the transformed Mg:Er:LiNbO_3_ crystals was studied in [44]. Mg-doped (0.5 and 1 mol%) near-stoichiometric LN crystals were prepared via Li-rich growth and VTE methods in [45]. Ru:Mg co-doped LN crystals were successfully grown using the Czochralski method in [46]. The Ru concentration was 0.02 mol%, and Mg had two concentrations: 4.0 and 6.0 mol%. In [47], the growth of Pr-Mg co-doped LN crystals using the Bridgman method is shown, and the optical properties of the crystals were studied. These are only a few examples showing the breadth of applications of LN:Mg crystals in modern science.

However, none of these works studied crystals co-doped with a metallic (Mg) and a non-metallic dopant (B). Therefore, an extremely interesting task in optical materials science is the study of LiNbO_3_:Mg:B crystals and the comparison of their structural and physicochemical characteristics with the characteristics of LiNbO_3_:Mg crystals. LiNbO_3_:Mg:B crystals are doped simultaneously with a non-photorefractive metallic dopant (Mg) and a non-metallic dopant (B). Both dopants reduce the effect of photorefraction, and B also increases the optical and compositional uniformiy of the LN crystal.

Two series of LiNbO_3_:Mg:B crystals grown from charges of various geneses were studied, and the charges were synthesized via different doping methods.

Two different methods for obtaining a doped LiNbO_3_:B:Mg charge were studied and described earlier in [48,49,50]. The first method is the solid-phase synthesis of a charge from the (Nb_2_O_5_):(Li_2_CO_3_):(MgO):(H_3_BO_3_) mixture. It also is called solid-phase (SP) doping. The second method is the sol–gel synthesis of the Nb_2_O_5_:B:Mg precursor, followed by the SP synthesis of the mixture (Nb_2_O_5_:B:Mg):(Li_2_CO_3_). It also is called homogeneous doping (HG). In cases of both double (Mg and B) and single doping (Mg) [17,51], HG doping helps to introduce approximately 25% more magnesium into the LiNbO_3_ crystal than SP doping at the same concentration of dopant in the melt, but no explanation for this fact was provided in [17,51].

The physicochemical features of the growth, structural, and optical characteristics of LiNbO_3_:Mg:B crystals are considered in this study. The crystals were grown from a Li_2_O-Nb_2_O_5_-MgO-B_2_O_3_ charge. The charge was obtained via SP and HG doping. Important tasks of this work are the search for physicochemical and structural reasons for the differences in the distribution coefficients (K_D_) of magnesium during the growth of LiNbO_3_:B:Mg and LiNbO_3_:Mg crystals from a HG- and SP-doped charge. The other task is to evaluate how the presence of boron influences the melt and the growth of LiNbO_3_:B:Mg crystals, their microstructure, and their compositional and optical uniformity. Comparative studies of the structures of LiNbO_3_:Mg and LiNbO_3_:Mg:B crystals obtained via SP and HG doping are performed using a full-profile XRD analysis. The structural characteristics of LiNbO_3_:Mg and LiNbO_3_:Mg:B crystals of different geneses are compared with the characteristics of a near-stoichiometric NSLN crystal.

## 2. Materials and Methods

Growing optically and compositionally uniform LiNbO_3_:B:Mg crystals with double doping is a non-trivial technological problem. Dopants have absolutely different (K_DMg_ ≈ 0.9–1.47 [17,51]; K_DB_ ≈ 1 × 10^−2^ [9]) distribution coefficients. Hence, the composition of the melt near the crystallization front during crystal growth can be simultaneously enriched in one doping component and depleted in the other or enriched in both doping components with different intensities. Thus, the composition of the doped crystal can significantly change during growth. This usually reduces its compositional and optical uniformity. To minimize such effects, it is necessary to adjust the parameters that are natural for crystal growth: the speeds of the rotation and pulling of the crystal, temperature gradients in the melt and growth zones, and various combinations of these parameters.

**Figure 1:**
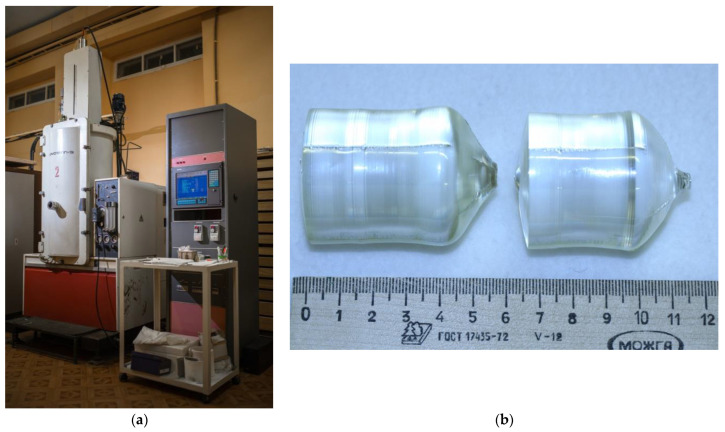
Kristall-2 growth setup for growing LN crystals via the Czochralski method (**a**); samples of as-grown LiNbO_3_:B:Mg crystals grown from a charge obtained via SP and HG doping (**b**).

Single crystals of LiNbO_3_:B:Mg and LiNbO_3_:Mg were grown from a platinum crucible ⌀85 mm under the conditions of a small (~3.0 deg/cm) axial gradient in the direction of the polar axis (Z-cut), a rotation speed of ~12 rpm, and a pulling speed of ~0.6 mm/h. The crystal growth rate was ~0.8 mm/h. The crystals were grown in a growth facility, a Kristall-2 (Voroshilovgradsky zavod electronnogo mashinostroeniya, Voroshilovgrad, USSR) equipped with an automatic crystal diameter control system, Figure 1a.

The design of the thermal unit was similar to the design used in [52]. Figure 1b shows samples of LiNbO_3_:B:Mg crystals grown from a charge obtained via SP and HG doping.

The growth of the LiNbO_3_:B:Mg and LiNbO_3_:Mg crystals was completed when crystal weights ≤ ~200–250 g were reached. About 20–25% of the total weight of the melt crystallized in this case. The parameters of the LiNbO_3_:B:Mg and LiNbO_3_:Mg crystal growth process were selected based on the need to obtain a flat crystallization front. The grown crystals had flat or slightly convex crystallization fronts (Figure 1b) and geometric dimensions of ⌀ ≈ 35–38 mm and the length of the cylindrical part L_c_ ≈ 35–40 mm.

The crystal melt was kept for 8–11 h before the initiation of growth under conditions of overheating by 180–200 °C relative to the melting point of LN (T_melt_ = 1263 °C) to homogenize the impurities in the melt. Thermal annealing of the LiNbO_3_:B:Mg crystal was carried out at 1230 °C in a growth setup for 15 h after growth, and it was then cooled at a rate of ~50 deg/h.

The LiNbO_3_:B:Mg and LiNbO_3_:Mg crystals were turned to a single domain state via high-temperature electrodiffusion annealing. A constant electric voltage was applied to the polar cuts of the crystal while it was cooling at a rate of 20 deg/h in the temperature range of ~1230–730 °C.

The NSLN crystal was grown via the HTTSSG (high-temperature top-seeded solution growth) method from a nominally pure granular charge of congruent composition with the addition of 5.5 wt% of high-purity K_2_O to the melt with a concentration of impurities at a level of <6 × 10^−4^ wt%. The crystal was grown in an air atmosphere from a platinum crucible ⌀75 mm under conditions of a relatively small (2 deg/cm) axial gradient in the direction of the polar axis (Z-cut) and at constant speeds of rotation (15 rpm) and displacement (~0.23 mm/h). The crystal growth rate was ~0.29 mm/h. The NSLN crystal was also grown in a Kristall-2 growth setup, Figure 1a. The NSLN crystal had a flat crystallization front and geometric dimensions: ⌀ ≈ 30 mm, length of the cylindrical part L_c_ ≈ 35 mm.

LiNbO_3_:B:Mg and LiNbO_3_:Mg crystals were grown from a congruent granular charge. Two series of LiNbO_3_:B:Mg crystals were grown. One series was grown from the charge obtained via HG doping. The other was grown from a charge obtained via SP doping. A series of LiNbO_3_:Mg crystals was grown from a charge obtained via SP doping; two LiNbO_3_:Mg crystals were obtained via HG doping. In the case of HG doping, boron and magnesium were introduced at the stage of the precipitation of niobium hydroxide from high-purity niobium-containing fluoride solutions. Then, high-temperature synthesis granulation of the Nb_2_O_5_:B:Mg-Li_2_CO_3_ mixture was carried out. A detailed technological scheme of the synthesis of the LiNbO_3_:B:Mg HG-doped charge can be found in [48]. In the case of SP doping, the synthesis granulation of the Li_2_CO_3_-Nb_2_O_5_-H_3_BO_3_-MgO mixtures was carried out [49]. Boron was added in the form of boric acid, H_3_BO, and magnesium in the form of magnesium oxide, MgO. SP doping included the synthesis granulation of the Li_2_CO_3_-Nb_2_O_5_-MgO mixture for a series of LiNbO_3_:Mg crystals and the HG doping–synthesis granulation of the Nb_2_O_5_:Mg-Li_2_CO_3_ mixture for two LiNbO_3_:Mg crystals. Magnesium was introduced at the stage of the precipitation of niobium hydroxide from high-purity niobium-containing fluoride solutions in the preparation of Nb_2_O_5_:Mg pentoxide. H_3_BO and high-purity MgO with a concentration of impurities at a level of <5 × 10^−4^ wt% were used.

Niobium pentoxide, Nb_2_O_5_ brand A, produced to Specifications No. 1763-025-00545484-2000 at Solikamsk Magnesium Works (Solikamsk, Russia), was used for charge synthesis. High-purity lithium carbonate, Li_2_CO_3_, with an impurity concentration of <3 × 10^−4^ wt% was also present in the charge. When the required amount of Li_2_CO_3_ was calculated to obtain a congruent LiNbO_3_ charge, the magnesium content in the charge was taken into account. The required amount of Li_2_CO_3_ was calculated without taking into account the content of boron in the charge due to the low concentration of the latter. The B concentration can be compared to the concentration of trace amounts of uncontrolled impurities. The synthesis granulation of each type of charge (Nb_2_O_5_:B:Mg-Li_2_CO_3_; Li_2_CO_3_-Nb_2_O_5_-H_3_BO_3_-MgO; Li_2_CO_3_-Nb_2_O_5_-MgO, and Nb_2_O_5_:Mg-Li_2_CO_3_) was carried out at a temperature of ~1235–1245 °C for 5 h. The heating rate of the mixtures was ~200 °C/h. As a result, a granulated charge with a high bulk density (~2.8–2.9 g/cm^3^) was obtained.

A series of four HG-doped LiNbO_3_:B:Mg crystals were grown from an HG-doped charge with a magnesium concentration of [MgO] = 3.4 mol% and [B] = 0.00857 wt% via a stepwise dilution of the initial melt. A nominally pure congruent LN charge was added to the melt remaining in the crucible after growing the previous crystal. This occurred after each LiNbO_3_:B:Mg crystal was grown. The studied range of the magnesium concentration in the melt was 2.5–3.4 mol%.

A series of four SP-doped LiNbO_3_:B:Mg crystals were grown from an SP-doped charge with a magnesium concentration of [MgO] = 3.66 mol% and [B] = 0.009 wt% via a stepwise dilution of the initial melt. The studied range of the magnesium concentration in the melt was 2.67–3.66 mol%.

A series of four SP-doped LiNbO_3_:Mg crystals were grown from an SP-doped charge with an initial magnesium concentration [MgO] = 5.2 mol% via a stepwise dilution of the initial melt. The studied range of the magnesium concentration in the melt was 3.4–5.2 mol%.

We also grew two HG-doped LiNbO_3_:Mg crystals with dopant concentrations in the crystals of 4.7 and 5.0 mol%, respectively.

To determine the impurity concentrations in the crystal after post-growth thermal annealing, wafers from the upper (conical—C_c_) and lower (bottom—C_b_) parts of the crystal boule were cut off. The wafer thickness was 0.8 mm. They were needed for the preparation of powder samples. The magnesium concentrations in the charge and crystals were determined via atomic emission spectrometry (AES) ICPE-9000 (Shimadzu, Japan, Kyoto, 2011) with an accuracy of 4 × 10^−3^ wt%, and the boron content was determined via ICP-MS with an accuracy of 1 × 10^−6^ wt%. XRD analyses of the charge and LiNbO_3_:B:Mg crystals were carried out on a diffractometer XRD-6100 (Shimadzu, Japan, Kyoto). The International Center for Diffraction Data (ICDD) electronic powder database was used for phase identification.

Table 1 shows the following data: the impurity compositions of the Nb_2_O_5_B:Mg precursor, LiNbO_3_:B:Mg, and the LiNbO_3_:B:Mg charges obtained via HG and SP doping. Spectral analysis methods determined the composition.

Plane-parallel crystalline wafers which were 1 mm thick in the Y- and Z-orientations were cut out from the LiNbO_3_:B:Mg and LiNbO_3_:Mg crystals to study their macro- and microstructures. The surfaces of the wafers were carefully polished. The study of the macro- and microstructure of the crystals was carried out via optical microscopy with a Thixomet image analysis system. The system includes an optical microscope, an Axio Observer.D1m (Carl Zeiss, Oberkochen, Germany), and software, Thixomet Standard (Thixomet, Saint-Petersburg, Russia), in bright field and differential interference contrast (DIC) modes. The Y- and Z-oriented crystalline wafers were etched in a mixture of mineral acids (HF + HNO_3_, 293 K, 20 h) before the study.

The optical transmission spectra of the LiNbO_3_:B:Mg crystals were studied using a spectrophotometer UVI-256 (LOMO, Sankt-Peterburg, Russia). Thoroughly polished Z-oriented crystalline wafers ~1 mm thick and boule fragments ~22 mm thick were used for the study.

Samples in the form of rectangular parallelepipeds 5 × 7 × 9 mm^3^ in size, with the largest size along the X-axis, were cut to record photoinduced light scattering (PILS) patterns and conoscopic patterns in widely diverging laser radiation beams from LiNbO_3_:B:Mg crystals. The edges of the parallelepipeds coincided in direction with the main crystallophysical axes (X, Y, Z). The faces of the parallelepipeds were carefully polished.

A Nd:YAG laser (MLL-100, Changchun New Industries Optoelectronics, Changchun, China, λ = 532 nm, I ~ 6.3 W/cm^2^) was used in the PILS and laser conoscopy experiments. During the PILS studies, the radiation scattered by the crystal fell on a semitransparent screen placed behind the crystal, and the entire process was recorded by a digital video camera. The laser beam was directed along the Y-axis, and the electric field strength vector E of the laser radiation was parallel to the polar axis of the crystal Z in the PILS experiments.

In the laser conoscopy studies, the sample was mounted on a movable two-coordinate optical stage. This helped to obtain many conoscopic patterns from different parts of the cross section of the sample. The conoscopic patterns were recorded on a translucent screen with a digital camera. A more detailed description of the methods for studying PILS and laser conoscopy in widely diverging laser beams, as well as block diagrams of the experimental setups, are presented in [52].

The main stages of the XRD analysis of the defective structures of the crystals were as follows:

- Obtaining a precise diffraction pattern; determining the size and shape of a unit cell with high accuracy;
- Refining the coordinates and parameters of the thermal motion of atoms;
- Calculating the site population factors;
- Establishing models for the incorporation of dopants into the lattice of LN crystals;
- Analyzing the oxygen packing distortions that occur when a dopant is introduced into the lattice.

The calculation of the profile characteristics of the obtained XRD patterns was performed via the Pauli method (the expansion of XRD patterns into the sum of the integral intensities). The Rietveld method (full-profile analysis) was used to refine the structural characteristics (atomic coordinates, thermal motion parameters, and site population factors). The Rietveld method is based on the construction of a theoretical XRD pattern according to a given model and its comparison with an experimental XRD pattern. In this case, the functional was minimized, which is the sum of the squared differences between the experimental and theoretically calculated scattering intensities at each point of the XRD pattern. Two software packages were used to solve the tasks in this study. The software packages implemented a full-profile analysis of the X-ray images via the Rietveld method: MRIA [53] and FULL PROF [54].

When we performed a full-profile analysis of the crystals under study, the X-ray patterns were recorded on a diffractometer, a DRON-6 (NPP Burevestnik, Sankt-Peterburg, Russia), with monochromatic CuKα radiation (CuK_α_ radiation, tube voltage—35 kV, current—20 mA) in the range of angles scattering 2θ from 3 to 145°. The reflection regions of the XRD pattern were captured with a small step in scattering angles for precision calculations via the full-profile analysis method. Therefore, the XRD pattern was divided into areas of band and background registration. Peak areas were recorded in more detail with a step of 0.02° and in the background area with a step of 0.2°. Control over the stability of the recording scheme was achieved while recording the XRD pattern. In addition, XRD patterns were taken repeatedly. The accuracy in determining the intensity at each point of the diffraction line was no less than 3%.

## 3. Results and Discussion

### 3.1. Physical and Chemical Features of LiNbO_3_:B:Mg and LiNbO_3_:Mg Crystals’ Growth

Table 2 shows the magnesium concentrations in the LiNbO_3_:B:Mg and LiNbO_3_:Mg crystals doped using various methods. The LiNbO_3_:B:Mg and LiNbO_3_:Mg crystals are labeled in the article based on Table 2. For example, sample B:Mg1-HG means LiNbO_3_:B:Mg crystal number 1 from Table 2, which was obtained via HG doping with a magnesium concentration of 4.2 mol%. Sample Mg4-SP means LiNbO_3_:Mg crystal number 4 from Table 2, which was obtained via SP doping with a magnesium concentration of 3.4 mol%, etc.

Such a strong discrepancy between the K_D_ values for the LiNbO_3_:B:Mg and LiNbO_3_:Mg crystals grown from an HG- and SP-doped charge requires an explanation. The explanation lies in the consideration of the chemical and physicochemical features of the synthesis of the charge and the growth of the crystals. The fundamental difference between the two methods of charge synthesis is the different doping sequence of the initial component (Nb_2_O_5_). This causes competition between lithium and a metal dopant (Mg) for octahedral positions in the LN structure when growing a crystal from a doped melt. Two processes simultaneously occur during the SP synthesis of the Nb_2_O_5_-Li_2_CO_3_-MgO-H_3_BO_3_ mixture: a lithium niobate phase based on Nb_2_O_5_ is formed, and this phase is doped with magnesium. In the case of HG, the sequence is different: first, the Nb_2_O_5_:B:Mg precursor is obtained via sol–gel synthesis and is then sintered with lithium carbonate (Li_2_CO_3_). The precursor is a mixture of two phases enriched with trace amounts of boron: magnesium niobate Mg_0.67_Nb_11.33_O_29_:B and niobium pentoxide Nb_2_O_5_:Mg:B. In HG, it is the sequence of chemical reactions in the Nb_2_O_5_:Mg:B-Mg_0.67_Nb_11.33_O_29_:B-Li_2_CO_3_ mixture that allows magnesium to occupy specific sites in the structure of one of the precursor phases (apparently, Mg_0.67_Nb_11.33_O_29_:B). These sites will become octahedral positions in the structure of the LiNbO_3_:B:Mg crystal, which will be occupied by Mg^2+^ cations after the formation of the lithium niobate monophase upon interaction with Li_2_CO_3_. In our opinion, this determines the significantly higher K_D_ values in the case of the HG-doped LN crystals compared to the SP-doped crystals, Table 2.

**Figure 2:**
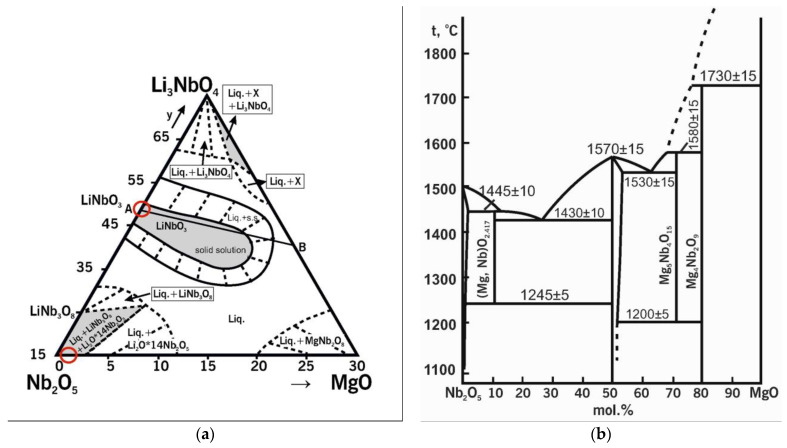
Part of the Li-Nb-Mg-O system: (**a**) isothermal cross-section at 1225 °C in the quasi-ternary state diagram Li_2_O-Nb_2_O_5_-MgO; (**b**) quasi-double state diagram of Nb_2_O_5_-MgO. The circles mark the regions of the initial compositions during doping.

Figure 2a shows the quasi-ternary state diagram of the Nb_2_O_5_-Li_2_O-MgO system [55]. The circles mark the regions of the initial compositions during doping, which are located on different sides of the phase triangle. For example, in case of the SP charge, the initial compound is Nb_2_O_5_ (LiNbO_3_); this oxide is located on the Nb_2_O_5_-Li_2_O side. In the case of the HG charge, the initial compound is a precursor consisting of two phases: Mg_0.67_Nb_11.33_O_29_ and Nb_2_O_5_:Mg; both phases are located on the Nb_2_O_5_-MgO side. The state diagram of Nb_2_O_5_-Li_2_O-MgO shows that the LiNbO_3_ compound has an extended region of homogeneity. This indicates the existence of ionic complexes in the melt in this concentration range with a certain structural–compositional difference. Figure 2b displays the quasi-double state diagram of Nb_2_O_5_-MgO [56]. The region of homogeneity of Nb_2_O_5_ on this diagram exists under the conditions of thermodynamic equilibrium. This region is located above 1100 °C and does not even reach ~1 mol% of MgO. A two-phase region is clearly observed in the concentration and temperature ranges under study. Other forms of magnesium niobate are formed instead of the MgNb_2_O_6_ phase (88-0708 ICDD card) in the absence of thermodynamic equilibrium in this concentration range. In our case, it was Mg_0.67_Nb_11.33_O_29_ (26-1218 ICDD card).

**Figure 3:**
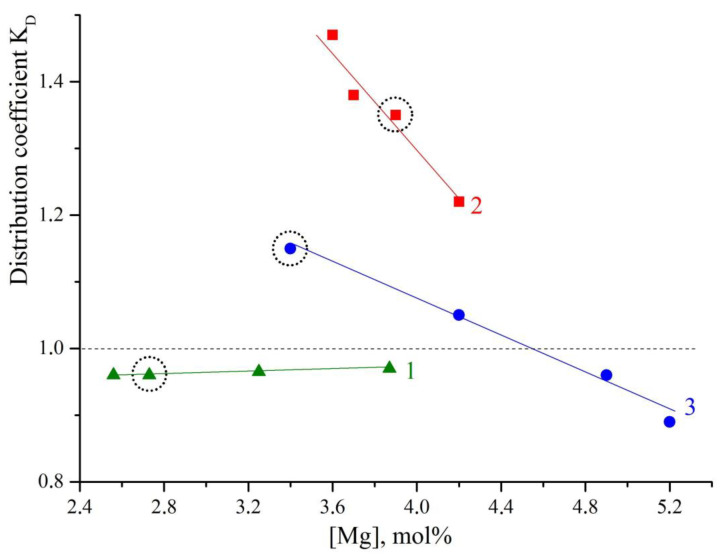
Dependence of K_D_ on magnesium concentration in LiNbO_3_:B:Mg crystals grown from SP-doped charge (1) and HG-doped charge (2); in LiNbO_3_:Mg crystals grown from SP-doped charge (3). Circles mark LiNbO_3_:B:Mg and LiNbO_3_:Mg crystals grown from melts with the same magnesium concentrations.

Figure 3 shows the dependence of K_D_ on the magnesium concentration in the LiNbO_3_:B:Mg and LiNbO_3_:Mg crystals. This dependence proves that the melt inherits the charge structure. Curve 1 in Figure 3 refers to the growth of LiNbO_3_:B:Mg crystals from the SP-doped charge. The curve shows that the K_D_ changes little; the coefficient gradually approaches the value of K_D_ = 1 as the magnesium concentration increases. Such a coefficient is most favorable for growing compositionally and optically uniform LiNbO_3_ crystals. Curve 2 in Figure 3 characterizes the growth of LiNbO_3_:B:Mg crystals from the HG-doped charge. The distribution coefficient in HG doped LiNbO_3_:B:Mg crystals, in general, is significantly higher than in SP doped LiNbO_3_:B:Mg crystals, Figure 3. In addition, the K_D_ sharply decreases over a very short concentration range for LiNbO_3_:B:Mg HG-doped crystals, Figure 3(2).

The K_D_ value is significantly lower for LiNbO_3_:Mg SP-doped crystals than for LiNbO_3_:B:Mg HG-doped crystals, Figure 3(2) and (3). The K_D_ = f([Mg]) dependence is also almost linear for LiNbO_3_:Mg SP-doped crystals. The K_D_ of the LiNbO_3_:Mg SP-doped crystals decreases much less sharply than the K_D_ of the LiNbO_3_:B:Mg HG-doped crystals; the K_D_ of the LiNbO_3_:Mg SP-doped crystals can be both lower and greater than 1. The K_D_ values of two LiNbO_3_:Mg HG-doped crystals also decreased with an increase in the concentration of magnesium, Table 2.

Thus, both the doping method and the presence of boron in the melt radically affect the physicochemical conditions of the crystallization of magnesium-containing LN crystals. Figure 3 vividly illustrates this effect: the circles mark LiNbO_3_:B:Mg and LiNbO_3_:Mg crystals grown from melts with the same magnesium concentrations; however, for these crystals, the charge geneses were different, and some of the crystals contained boron, while others did not.

Figure 3 clearly shows that in the presence of boron, K_D_ approaches unity and is almost independent of the magnesium concentration in the melt. This means that boron significantly increases the compositional uniformity of LiNbO_3_:B:Mg crystals, at least in the case of SP doping.

**Figure 4:**
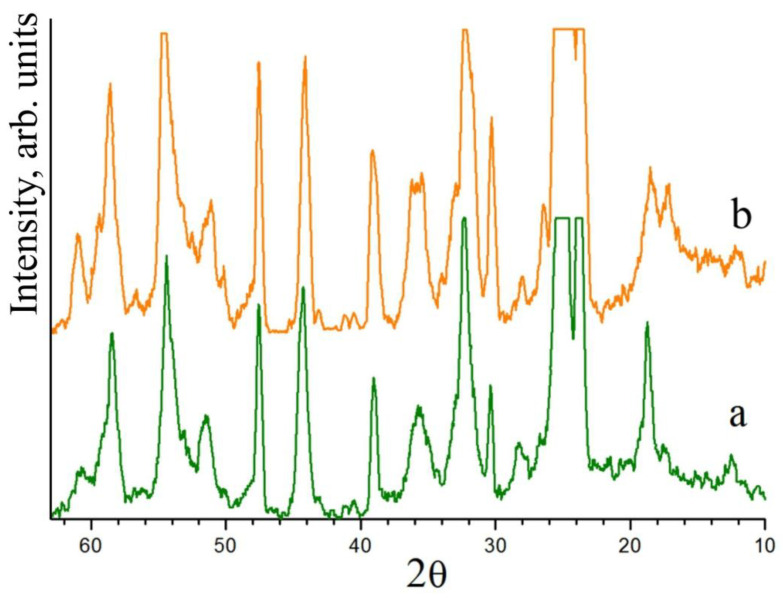
XRD patterns of precursors Nb_2_O_5_:B:Mg (a) and Nb_2_O_5_:Mg (b). Precursors were obtained via the sol–gel method and calcined at 1000 °C.

Boron is a strong complexing agent, and its chemical properties narrow the variety of ionic complexes in the melt and increase the region of homogeneity of LiNbO_3_:Mg in the Li_2_O-Nb_2_O_5_-MgO quasi-ternary system. At the same time, the effect of boron on the phase composition of the Nb_2_O_5_:B:Mg precursor in a solid state has not been recorded. The polyphase composition of the Nb_2_O_5_:Mg precursor obtained via the sol–gel method was analyzed in detail in [57]. The XRD pattern of this Nb_2_O_5_:Mg precursor does not differ from the XRD pattern of the Nb_2_O_5_:B:Mg precursor obtained via the sol–gel method in neither the number of reflections nor in their intensities, Figure 4. Boron actively affects the molten state of the LN charge, but it hardly affects the structure of the solid state of the initial boron-containing components.

### 3.2. Study of the Defect Structure of LiNbO_3_:B:Mg Crystals via Full-Profile Analysis Methods

**Figure 5:**
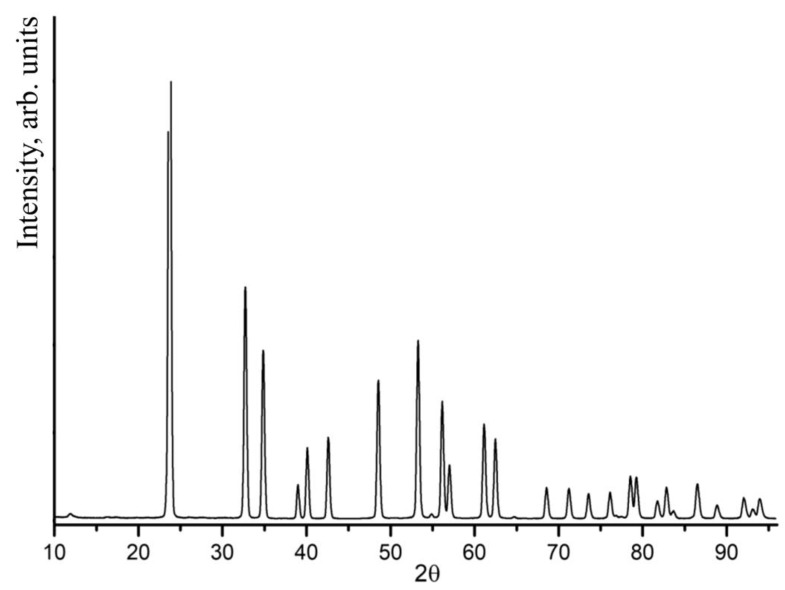
XRD pattern of LiNbO_3_:Mg:B ([Mg] = 3.6 mol%) crystal.

All recorded XDR patterns of LiNbO_3_:Mg:B crystals (both SP- and HG-doped) corresponded to the XRD pattern of LN with the space symmetry group R3c (ferroelectric phase). The most intense reflection in the XRD patterns of the crystals under study appears at a scattering angle of 23.7°. Figure 5 shows an XRD pattern of a B:Mg4- HG ([Mg] = 3.6 mol%).

Table 3 shows the refined values of coordinates (*x/a*, *y/b*, *z/c*) of the atoms, the periods (*a*, *c*) of the unit cell, the site population factors (G) in the lattice, and the values of uncertainty factors for two HG-doped LiNbO_3_:Mg:B crystals. The position of boron in the structure was not determined due to its low concentration of about ~10^−4^ wt%.

The unit cell periods are *a* = 5.1428 Å, *c* = 13.8443 Å for a NSLN crystal. HG doping of the LN crystal with magnesium and boron increases the unit cell periods in comparison with the corresponding data for the NSLN crystal. The XRD data in Table 3 show that the dopant (Mg) occupies a regular site of lithium and an empty oxygen octahedron in the case of the HG-doped LiNbO_3_:Mg:B crystals, and the vacant octahedron (V) population factor is higher than the factor of the lithium site in both studied samples. In addition, when the concentration of magnesium increases, the number of niobium defects increases: Nb_Li_, Nb_V_, and V_Nb_.

Table 4 shows the refined values of the atom coordinates (*x/a*, *y/b*, *z/c*), the periods of the unit cell (*a*, *c*), the site population factors (G) in the lattice, and the values of the R-factors for the two studied SP-doped LiNbO_3_:Mg:B crystals.

The values of the unit cell periods in the SP-doped LiNbO_3_:Mg:B crystals are also higher than in the NSLN crystal. In contrast to the HG-doped LiNbO_3_:Mg:B crystals, magnesium occupies only the lithium site. The number of niobium defects localized in vacant octahedra (Nb_V_) noticeably decreases in the SP-doped LiNbO_3_:Mg:B crystals. Nb_V_ defects are almost absent, and the population factor $G_{{\mathrm{Nb}}_{V}}$ is only 0.004 in the B:Mg1-SP sample with the highest magnesium content (C(Mg) = 3.87 mol%). The obtained result shows that the degree of ordering of the cationic sublattice is higher in this sample than in the other considered samples of LiNbO_3_:Mg:B crystals. The excess charge of magnesium in the lattice is compensated for by the formation of niobium vacancies in both samples of SP-doped LiNbO_3_:Mg:B crystals. And there are no lithium vacancies. The antisite defect niobium in the lithium site (Nb_Li_) is observed in all studied HG- and SP-doped LiNbO_3_:Mg:B crystals, Table 3 and Table 4.

Thus, the XRD studies explain the significant increase in the K_D_ in the HG-doped LiNbO_3_:Mg:B crystals compared to the SP-doped LiNbO_3_:Mg:B crystals from the point of view of the point defect structures of the crystals. For example, magnesium occupies the regular site of lithium (Mg_Li_) and the vacant oxygen octahedron (Mg_V_) in the HG-doped LiNbO_3_:Mg:B crystals. This is because the crystal has inherited the precursor’s structure; in this case, the precursor was a mixture of Nb_2_O_5_:Mg:B-Mg_0.67_Nb_11.33_O_29_:B phases.

The Me-O (Me: Nb, Li, Mg) distances in the corresponding oxygen octahedra were calculated from the refined values of the atomic coordinates and the unit cell periods of the LiNbO_3_:Mg:B crystals and the NSLN crystal. Table 5 shows the obtained data.

The degree of distortion of the LiO_6_ lithium octahedron in the LiNbO_3_:Mg:B crystals increases in the case of HG doping, i.e., the difference between the long and short Li-O distances increases compared to the data for the NSLN crystal. Among the studied crystals, the difference between the short and long distances in the lithium octahedron, 0.141 Å, is the smallest in the SP-doped LiNbO_3_:Mg:B crystal with a magnesium concentration of 3.25 mol%.

The degree of distortion of a regular niobium octahedron increases when the concentration of magnesium in an HG-doped LiNbO_3_:Mg:B crystal increases. When the concentration of magnesium is 3.6 mol%, the difference between short and long Nb-O distances is 0.26 Å. This roughly corresponds to the distance in the NSLN crystal. When the magnesium concentration is 4.2 mol%, this value increases to 0.371 Å. The opposite situation is observed in SP-doped LiNbO_3_:Mg:B crystals.

The incorporation of magnesium into the lithium octahedron (Mg_Li_) does not lead to its strong distortion in SP-doped LiNbO_3_:Mg:B samples. And when magnesium enters the regular lithium sites, there are noticeable changes in the bond lengths in the Mg_Li_O_6_ octahedron in the HG-doped LiNbO_3_:Mg:B samples compared to the corresponding data for the regular LiO_6_ octahedron.

Thus, the structural states of LiNbO_3_:Mg:B crystals noticeably change depending on the technology used to obtain them. Magnesium occupies regular lithium sites (Mg_Li_) and regular vacant octahedra (Mg_V_) in HG-doped LiNbO_3_:Mg:B samples and only lithium sites (Mg_Li_) in SP-doped samples. The entry of magnesium into the lithium site noticeably changes the Mg-O distances in the corresponding Mg_Li_O_6_ octahedra in HG-doped LiNbO_3_:Mg:B crystals. Co-doping LiNbO_3_ with magnesium and boron increases the unit cell periods in comparison with the NSLN crystal regardless of the doping method. And the SP-doped LiNbO_3_:Mg:B crystal with a magnesium concentration of 3.87 mol% contains the least number of intrinsic defects among all the studied LiNbO_3_:Mg:B crystals.

To evaluate the effect of boron on the structure of an LiNbO_3_:Mg:B crystal, the defect structures of one SP-doped LiNbO_3_:Mg crystal and two HG-doped LiNbO_3_:Mg crystals were studied via a full-profile XRD analysis. The development of models for the nature of the location of intrinsic defects in the LN lattice [16,58] is possible on the basis of these data.

Two HG-doped LiNbO_3_:Mg crystals with magnesium concentrations of 5.0 and 4.74 mol% (Mg1-HG and Mg2-HG) and one SP-doped LiNbO_3_:Mg crystal with a magnesium concentration of 4.9 mol% (Mg2-SP) were studied. Table 6 presents the values of the unit cell periods of the LiNbO_3_:Mg crystals refined via the of full-profile analysis method.

The obtained data show that the values of the period *c* in all the studied samples of LiNbO_3_:Mg crystals of different geneses almost coincide and are higher by ~0.02 Å than that of the NSLN crystal. The values of the period *a* are also higher than in NSLN. The values of both periods of the unit cell in all variants of doping of LiNbO_3_:Mg crystals are close and are higher than in the NSLN crystal. This agrees with the results for LiNbO_3_:Mg:B crystals (HG and SP doping, Table 3 and Table 4).

The results of refinement of the structural characteristics of the studied Mg-doped LN crystals are shown in Table 7.

Table 7 shows that the population coefficient of niobium site is approximately the same—G~0.91—and niobium vacancies appear in all the samples under study. Some of the niobium atoms occupy vacant octahedron and lithium sites. The total occupation of these sites by niobium is ~0.038 regardless of the doping method. Most of the niobium is located in vacant octahedra in the Mg1-HG and Mg2-HG samples. The dopant concentrations in these samples are 5.0 and 4.74 mol%, respectively. And most of the niobium atoms occupy lithium sites, forming Nb_Li_ antisite defects in the Mg2-SP sample.

Magnesium occupies both vacant octahedra and lithium sites in the HG-doped LiNbO_3_:Mg crystals, Table 7. Magnesium occupies lithium sites and vacant octahedra in HG-doped LiNbO_3_:Mg:B crystals, Table 3. The HG-doped LiNbO_3_:Mg and LiNbO_3_:Mg:B crystals are similar in this, Table 3 and Table 7. Thus, no significant effect of boron on the features of the defect structures of the magnesium-containing HG-doped LN crystals is observed.

However, the defect structure is quantitatively different for SP-doped LiNbO_3_:Mg and LiNbO_3_:Mg:B crystals. In SP-doped LiNbO_3_:Mg:B crystals, all magnesium cations occupy lithium sites, forming Mg_Li_ defects with population factors ~0.035–0.039 (Table 4). In SP-doped LiNbO_3_:Mg crystals, magnesium cations also localize in lithium octahedra, forming Mg_Li_ defects but with a population more than 1.5 times higher, ~0.06, Table 7. This difference in the defect structures of the SP-doped LiNbO_3_:Mg and LiNbO_3_:Mg:B crystals is due to the structure-forming effect of boron in the melt.

Thus, the presence of boron in the melt during the preparation of LiNbO_3_:Mg:B crystals significantly changes their structural state compared to LiNbO_3_:Mg crystals. Different types of structural defects and distortions of the structure octahedra in LiNbO_3_:Mg:B and LiNbO_3_:Mg crystals unambiguously lead to differences in their physical characteristics.

### 3.3. Study of the Macro- and Microstructures of LiNbO_3_:B:Mg Crystals of Different Geneses

The method of obtaining a series of crystals with different dopant concentrations in a single technological cycle must be taken into account when analyzing the influence of the features of technology on the practically important characteristics of LiNbO_3_:B:Mg crystals of different geneses. The complication of the melt structure arises and sequentially accumulates in the case of the dilution method during the growth of a series of LiNbO_3_:B:Mg crystals. The spectrum of ionic complexes in the melt at the maximum dopant concentration differs from that at the minimum concentration. If each crystal were grown from a newly obtained melt, then in the lower limit of the dopant content, the melt would not contain complexes that are present in the upper limit of the concentrations of the range under study: they appear at a higher dopant concentration. In our case, when growing the first crystal, the dopant concentration was at a maximum. Thus, the spectrum of ionic complexes in the melt differs from that at the minimum concentration, when the last crystal of the series was grown.

When the dopant concentration is reduced via dilution, the melt inherits a certain amount of all variants of complexes corresponding to thermodynamic equilibrium, including ionic complexes inherent from the melt with the maximum concentration of the dopant. This increases the defectiveness of LiNbO_3_:B:Mg crystals.

**Figure 6:**
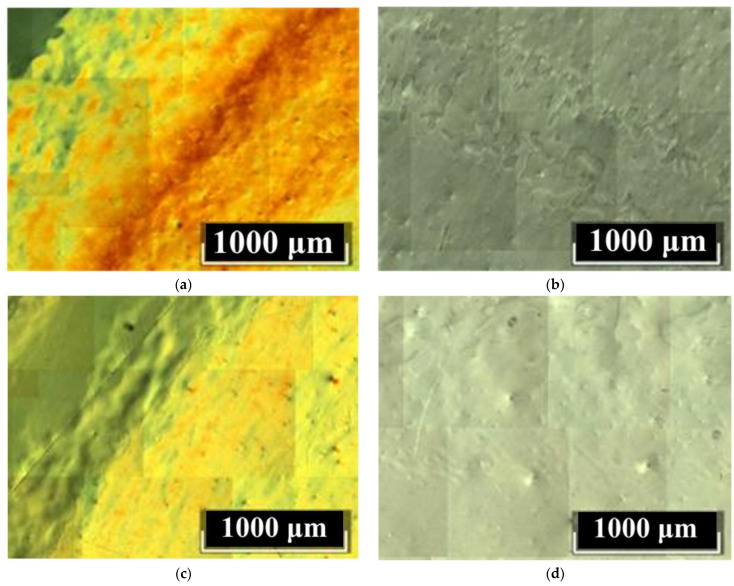
DIC images of LiNbO_3_:B:Mg crystals after thermal treatment: B:Mg4-HG (**a**) and B:Mg1-HG (**c**); B:Mg2-SP (**b**) and B:Mg3-SP (**d**). Z-cut.

However, the investigated LiNbO_3_:B:Mg crystals have sufficiently high structural and optical uniformities, at least those obtained via SP doping. Figure 6 shows DIC images of Z-cuts of LiNbO_3_:B:Mg crystals after thermal treatment that are typical for HG (a) and SP (b) doping.

The images in Figure 6 show the optical density deviations of the LiNbO_3_:B:Mg crystals. Moreover, deviations are manifested both at the micro- and macrolevels. At the macrolevel, the deviation is manifested as different pseudo-colors in the DIC image, Figure 6a,c. Such a defect is usually eliminated by another long-term, high-temperature heat treatment of the LiNbO_3_:B:Mg crystal. At the microlevel, the crystal’s non-uniformity appears as bumps or luminous dots on the DIC image. This is actually the same defect which, depending on the choice of focal length, manifests itself differently on the DIC image, Figure 6. This defect is present in all studied LiNbO_3_:B:Mg crystals regardless of the doping method and dopant concentration. However, the number of such microdefects in the SP-doped LiNbO_3_:B:Mg crystals is significantly lower than in the HG-doped LiNbO_3_:B:Mg, Figure 6. In addition, deviations in optical density are almost absent from the SP-doped LiNbO_3_:B:Mg crystals in contrast to the HG-doped LiNbO_3_:B:Mg crystals, compare Figure 6a,c and Figure 6b,d.

The B:Mg4-HG crystal is the most defective sample among the investigated LiNbO_3_:B:Mg crystals. It has a minimum dopant concentration of ~3.6 mol% in a series of HG-doped crystals, Figure 6a. The dilution technology used when growing a series of LiNbO_3_:B:Mg crystals is precisely the reason for this.

The melt of the LiNbO_3_:B:Mg crystals contains a strong complexing agent: boron. This feature is worth discussing separately. Boron almost does not incorporate the LiNbO_3_:B:Mg crystal but fundamentally changes the structure of the melt and also increases its viscosity with each crystal grown. This changes the rates of the diffusion processes and convective currents. Local regions of various types of nonequilibrium states are created in the melt. Even the dilution of the melt changes its composition from the initial one, it increases the concentration of boron due to the very small distribution coefficient of the dopant. Therefore, as the last crystal in the series, the B:Mg4-HG crystal is more defective than the previous LiNbO_3_:B:Mg HG-doped crystals from this growth cycle.

DIC images of the surfaces of the SP-doped Z oriented LiNbO_3_:B:Mg crystals have only a very slight macro-non-uniformity. The macro-non-uniformity is located only at the edges of the wafers. These crystals also contain a slight micro-non-uniformity in the form of luminous dots over the entire area of the samples, Figure 6b,d. This happens for two reasons: first, the two-phase SP-doped precursor Nb_2_O_5_:B:Mg does not inherit the structure, as in the case of HG doping; second, the K_D_ is close to unity, Table 2. In the studied concentration range of the LiNbO_3_:Mg,B SP-doped charge, this creates a more uniform and equilibrium structure of the melt. Accordingly, the imperfections of the crystals are relatively small.

**Figure 7:**
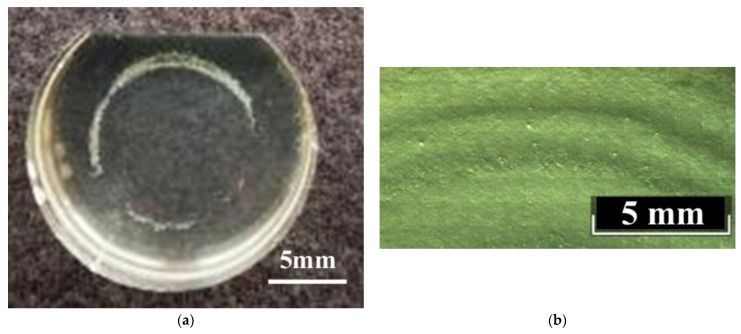
Macrostructure of Z-cut of B:Mg1-HG (**a**) and B:Mg4-SP (**b**) crystals. Image (**a**) was obtained in a bright field; image (**b**) was obtained by the DIC.

All studied LiNbO_3_:Mg,B crystals have weakly expressed growth rings (stripes) of irregular arrangements and lengths on Z-cuts, Figure 7. Such growth rings on the Z-cuts are usually observed in REE-doped LN crystals, but the rings are much clearer in those cases [59]. Growth rings are caused by convective flows in the melt [59]. The weak prominence of growth bands in LiNbO_3_:Mg,B crystals is related to the lower intensity of the convective flows. This is due to boron, which noticeably increases the viscosity of the melt, Figure 7.

**Figure 8:**
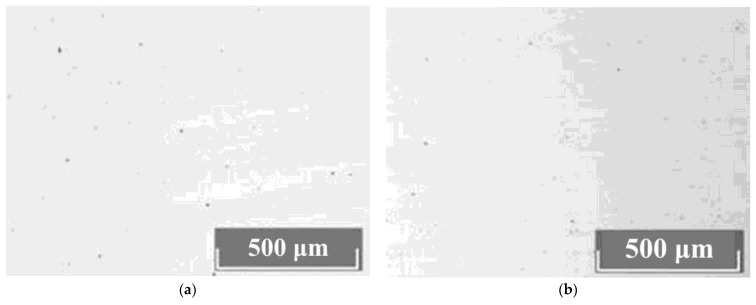

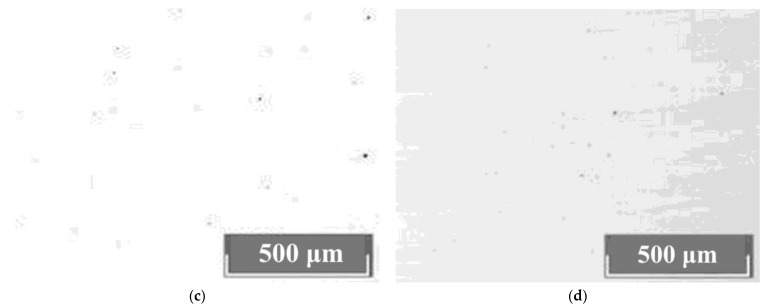
Microstructures of Y-cut LN crystals: Mg,B1-SP (**a**); Mg,B4-SP (**b**); Mg,B1-HG (**c**); Mg,B4-HG (**d**).

The Y-cuts of LiNbO_3_:Mg,B crystals are completely free of stripes (Figure 8) despite the fact that such stripes are quite usual for the majority of LiNbO_3_:Mg crystals of both HG and SP doping [17,60]. The absence of stripes on the Y-sections is an important positive consequence of the presence of a strong complexing agent in the melt: boron. The potential for the commercial use of LiNbO_3_:Mg for laser radiation converting is noticeably reduced if there are difficult-to-remove stripes in the crystal in the direction perpendicular to the growth axis.

**Figure 9:**
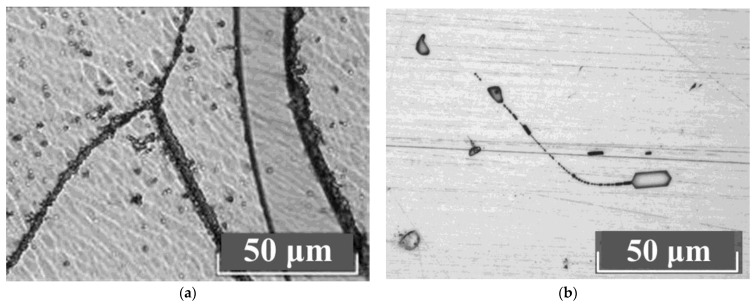

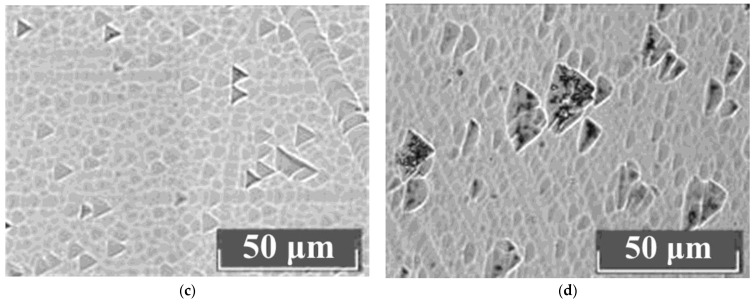
Microstructures of Z-cut LN crystals: Mg,B4-HG (**a**); Mg,B2-HG (**b**); Mg,B1-SP (**c**); Mg,B4-SP (**d**).

The study of the crystals’ microstructures via optical microscopy showed that all LiNbO_3_:Mg:B crystals have a basic block substructure (grid). Additional defects in the forms of clusters of triangular microdomains of various configurations are always present against the grid background. The density distribution of clusters is different, Figure 9. The boundaries of individual elements of the block structure are actually low-angle boundaries which are formed in a crystal by dislocations [61,62]. Thus, the boundaries of the blocks are dislocation structures in which internal stresses and defects are released.

Dislocation grids and lines appear at points at which mechanical and thermal stresses emerge in the HG-doped LiNbO_3_:Mg crystals, Figure 9a,b. Among the HG-doped LiNbO_3_:Mg,B crystals, the Mg,B4-HG crystal has the least uniform microstructure, Figure 9a. The microstructure of the Mg,B4-HG crystal differs from all the other studied LiNbO_3_:Mg,B crystals: it contains aggregates of blocks (substructure) with different preferred orientations, Figure 9a. This increases local mechanical stresses, and, as a consequence, microcracks form in the crystal. Such microcracks are intense sinks of dislocations, Figure 9a. Similar mass manifestations of dislocation structures are absent from SP-doped LiNbO_3_:Mg,B crystals, Figure 9c,d.

The most uniform microstructure was recorded in the Mg,B1-SP crystal ([Mg] = 3.8 mol%), Figure 9c. The most uniform structure among the HG-doped crystals was recorded in Mg,B2-HG crystal ([Mg] = 3.9 mol%). Thus, crystals with [Mg] = 3.9 mol% are the most uniform among the LiNbO_3_:Mg,B crystals doped via both SP and HG methods.

### 3.4. Optical Characteristics of LiNbO_3_:Mg,B Crystals of Different Geneses

**Figure 10:**
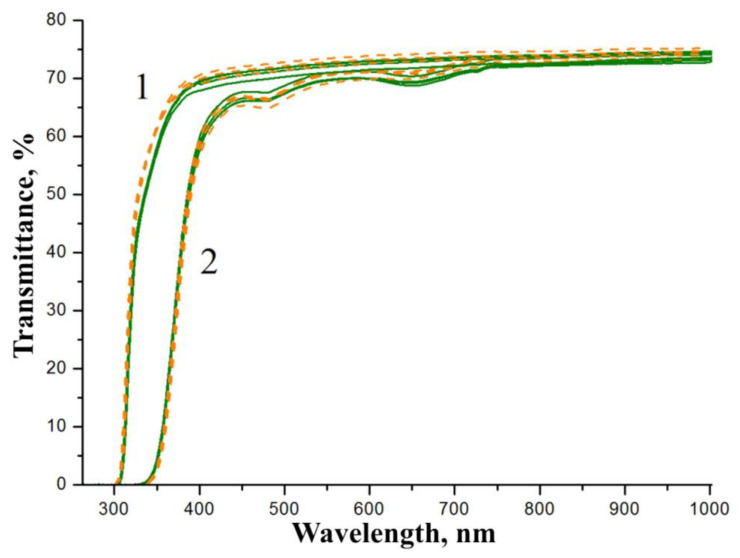
Transmission spectra of LiNbO_3_:Mg,B crystals: Mg,B1-SP (dashed line) and Mg,B2-HG (solid line). The crystalline wafers are ~1 mm thick (1) and the fragments of crystalline boules are ~22 mm thick (2).

Figure 10 demonstrates transmission spectra of samples of the SP- and HG-doped LiNbO_3_:Mg,B crystals. The shapes of the transmission spectra of SP- and HG-doped LiNbO_3_:Mg,B crystal generally do not depend on the doping method, but the shape changes slightly with a significant change in the thickness of the plates.

The transmission spectra reflect information about the optical characteristics of the crystal along the entire path of the light beam. Thus, an increase in the thickness of the sample under study reveals elements of the defective structure of the crystal which are not observed in thin wafers, Figure 10. The transmission level of a 1 mm thick wafer is slightly higher than that of a fragment of a crystalline boule with a thickness of ~22 mm, Figure 10. In addition, the large thickness of the samples and the “accumulated defects” lead to the appearance of very weak absorption bands near ~481 and ~652 nm, Figure 10(2). The bands are usually associated with the absorption of polarons and bipolarons [8]. Absorption bands near ~480 and ~650 nm are inherent in all LN crystals doped with a metal (Zn or Mg) via the HG method regardless of the concentration and type of dopant [61]. However, ~480 and ~650 nm absorption bands are absent from all LN crystals doped with a metal (Zn or Mg) via the SP method [61]. This result revealed one more effect of boron traces on LiNbO_3_:Mg,B crystals: weak absorption bands near ~481 and ~652 nm are observed for both SP- and HG-doped LiNbO_3_:Mg,B crystals, Figure 10(2).

**Figure 11:**
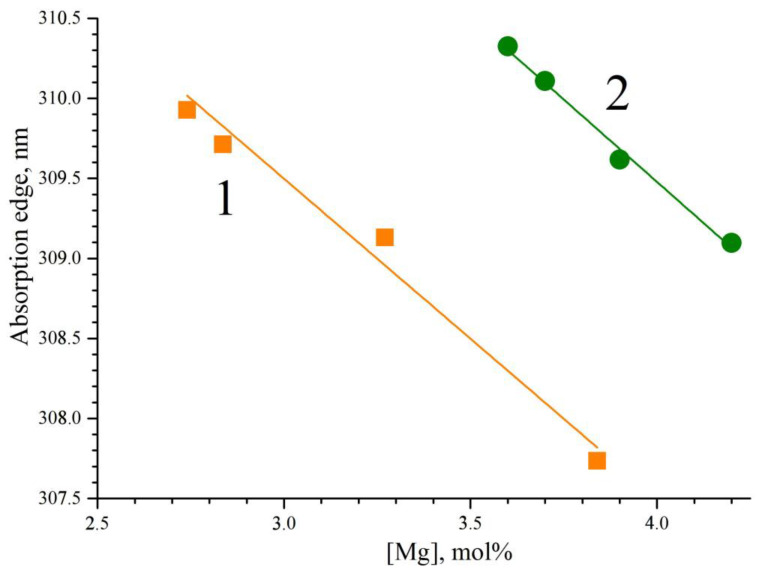
Dependence of the absorption edge on the concentrations of magnesium in LiNbO_3_:Mg,B crystals: SP-doped (1); HG-doped (2). Thickness of crystalline wafers ~1 mm.

Figure 11 demonstrates the dependence of the absorption edge on the concentration of magnesium in the LiNbO_3_:Mg,B crystals. The absorption edge shifts to the short-wavelength region with an increase in the magnesium concentration, Figure 11. The absorption edge of the SP-doped LiNbO_3_:Mg,B crystals is slightly (~3.5 nm) shifted to the region of short wavelengths when compared with the HG-doped crystals, Figure 11. This means that there are somewhat fewer charged electronic structural defects in the SP-doped crystals than in the HG-doped LiNbO_3_:Mg,B crystals. This result correlates with the data on the study of the defective structure of crystals using the Rietveld method, Table 3 and Table 4. In LiNbO_3_:Mg crystals, there is a concentration threshold after which the photorefraction effect is strongly suppressed. Due to [8], the threshold is located at ~5.5 mol% of MgO. The trace amounts of boron also influence the LiNbO_3_:Mg,B crystals here: their photorefractive sensitivity is noticeably suppressed at a much lower concentration of magnesium.

**Figure 12:**
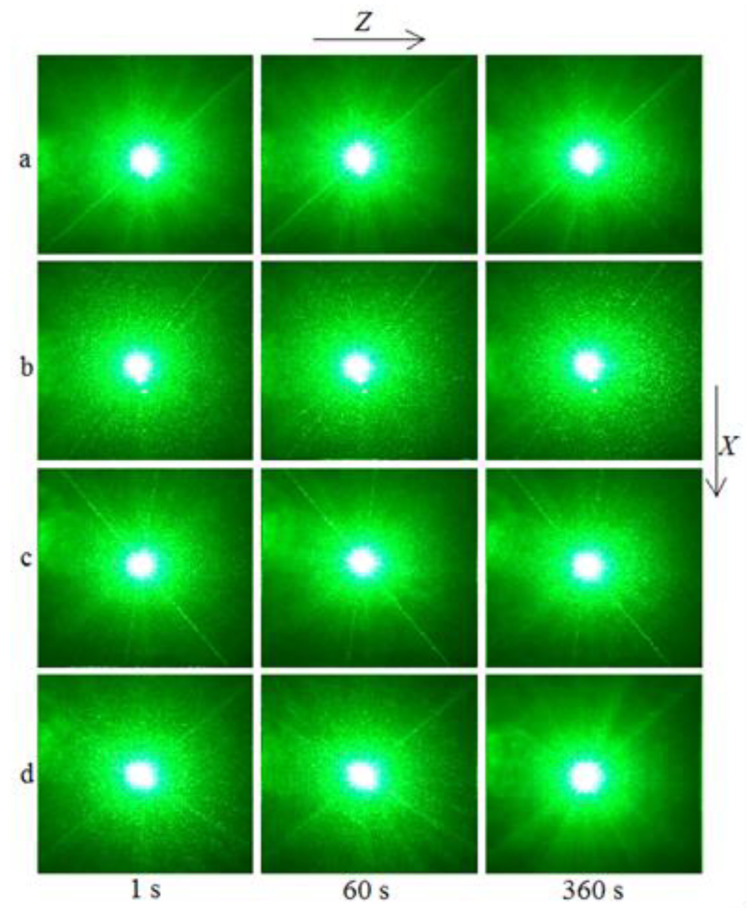
PILS patterns of SP-doped LiNbO_3_:Mg,B crystals: [Mg] = 2.56 (**a**), 2.73 (**b**), 3.25 (**c**), and 3.87 (**d**) mol%. λ = 532 nm. I~6.3 W/cm^2^.

**Figure 13:**
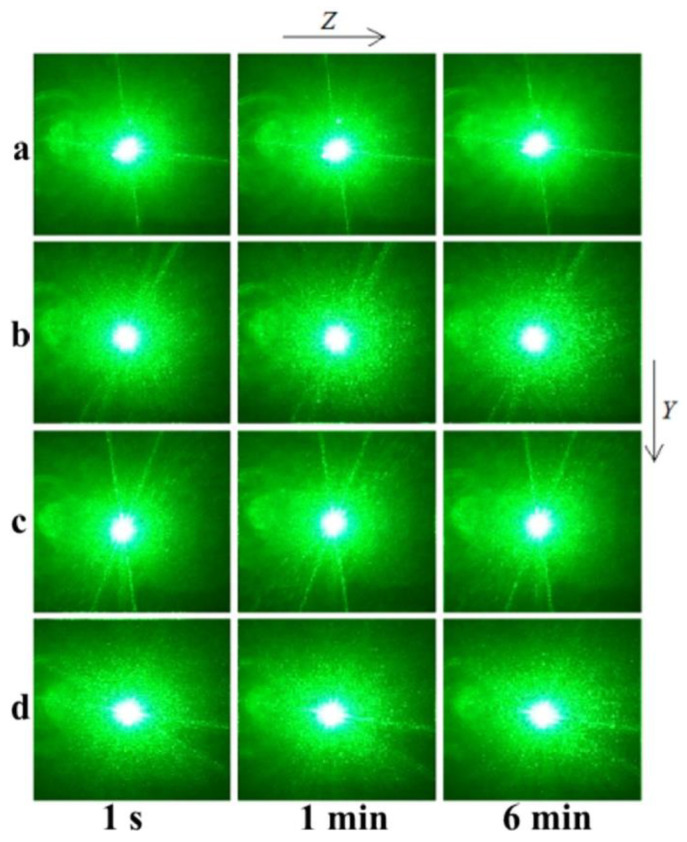
PILS patterns of HG-doped LiNbO_3_:Mg,B crystals: [Mg] = 3.6 (**a**), 3.7 (**b**), 3.9 (**c**), and 4.2 (**d**) mol%. λ = 532 nm. I~6.3 W/cm^2^.

The PILS patterns of the SP- and HG-doped LiNbO_3_:Mg,B crystals are shown in Figure 12 and Figure 13.

The photorefractive effect in LiNbO_3_:Mg,B crystals is suppressed at magnesium concentrations as low as ([MgO] = 2.56 mol%) for SP-doped crystals and ([MgO] = 3.6 mol%) for HG-doped crystals, Table 2, Figure 12 and Figure 13. For all studied LiNbO_3_:Mg,B crystals, no destruction of the laser beam is observed, Figure 12 and Figure 13. That is, even at a relatively high intensity of the exciting radiation (I~6.3 W/cm^2^), there is no photorefractive response, and the PILS indicatrix is not revealed. Only circular scattering by static structural defects is observed. The scattering pattern does not change with time and retains a circular shape throughout the experiment, Figure 12 and Figure 13.

**Figure 14:**
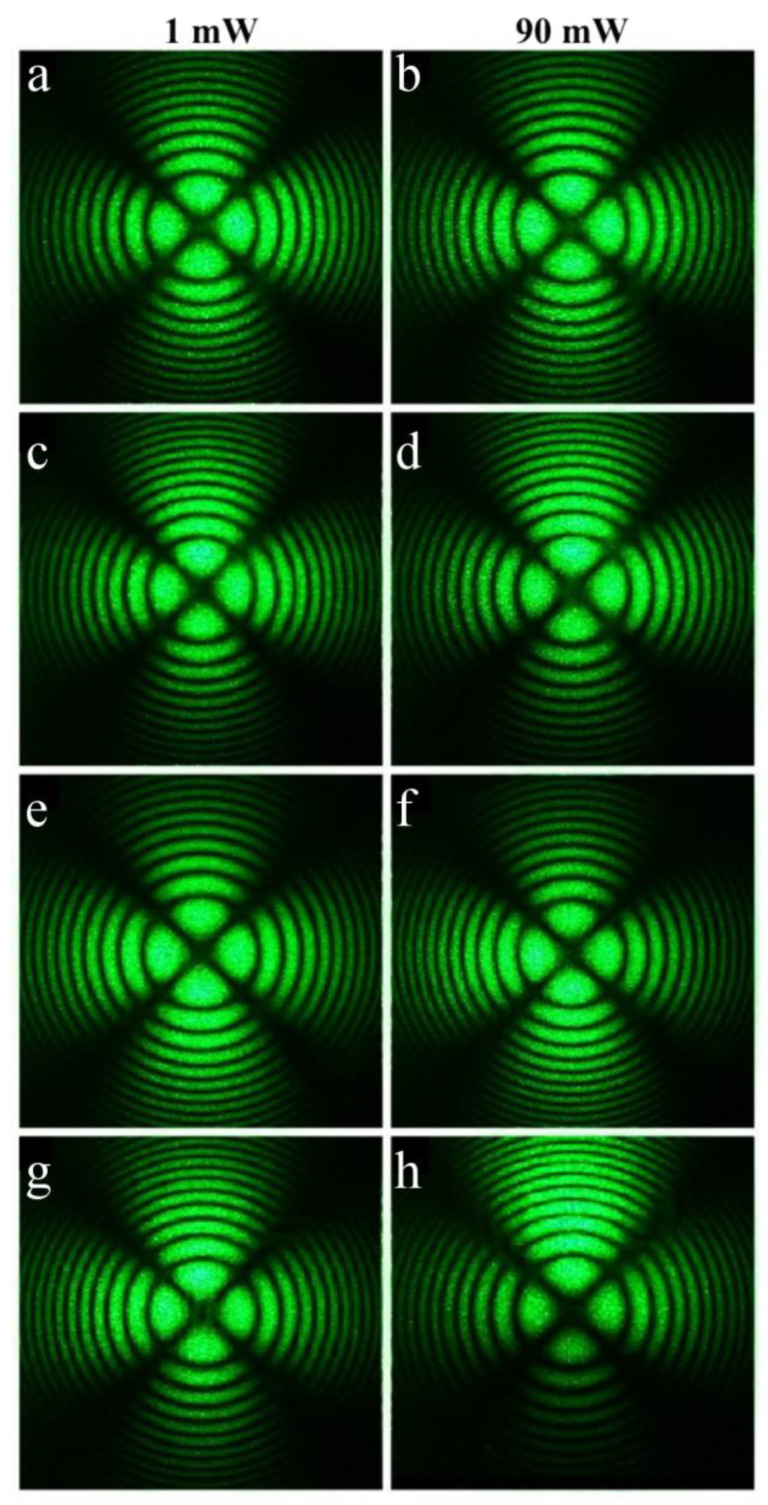
Conoscopic patterns of SP-doped LiNbO_3_:Mg,B samples: [Mg] = 3.87 (**a**,**b**), 3.25 (**c**,**d**), 2.73 (**e**,**f**), 2.56 (**g**,**h**) mol%. Wafer thickness ~3 mm. λ = 532 nm. P = 1 and 90 mW.

The SP- and HG-doped LiNbO_3_:Mg,B crystals were also studied via conoscopy. Conoscopic patterns confirm the high optical and structural uniformity of the SP-doped LiNbO_3_:Mg,B crystals, Figure 14. SP-doped LiNbO_3_:Mg,B crystals in general have a uniform microstructure, Figure 9c,d. In addition, the magnesium distribution coefficient in these crystals is close to unity, Table 2. This predetermines the high compositional uniformity of the crystal along the growth axis.

The conoscopic patterns of all SP-doped LiNbO_3_:Mg,B samples correspond to the standard conoscopic patterns of uniaxial crystals. A circular symmetry is observed, the black contrast Maltese cross retains its integrity in the center of the field of view, and the isochromes are concentric circles centered at the exit point of the optical axis, Figure 14. The most uniform microstructure among the SP-doped LiNbO_3_:Mg,B samples was recorded for the Mg,B1-SP crystal ([Mg] = 3.87 mol%), Figure 9c. Studies using laser conoscopy unequivocally confirm this. The highest-quality conoscopic patterns, which do not contain any defects, were recorded specifically for the Mg,B1-SP crystal ([Mg] = 3.87 mol%), Figure 14a,b. The structural uniformity of crystals in general decreases when using the dilution method in a single technological cycle; thus, the Mg,B4-SP crystal has somewhat less perfect conoscopic patterns, Figure 14g,h. The conoscopic patterns of the Mg,B4-SP crystal reveal small anomalies. The branches of the Maltese cross have anomalies in the form of a weakly expressed additional interference structure against the background of the main conoscopic pattern, and the Maltese cross is very slightly deformed, Figure 14g,h. In general, the conoscopic patterns of all studied SP-doped LiNbO_3_:Mg,B crystals correspond to an optically uniaxial crystal; the patterns do not show signs of anomalous optical biaxiality, Figure 14. Additional anomalies do not appear in the conoscopic patterns of the SP-doped LiNbO_3_:Mg,B crystals with increasing laser radiation power (Figure 14) since there is no photorefractive response in these crystals, Figure 12.

**Figure 15:**
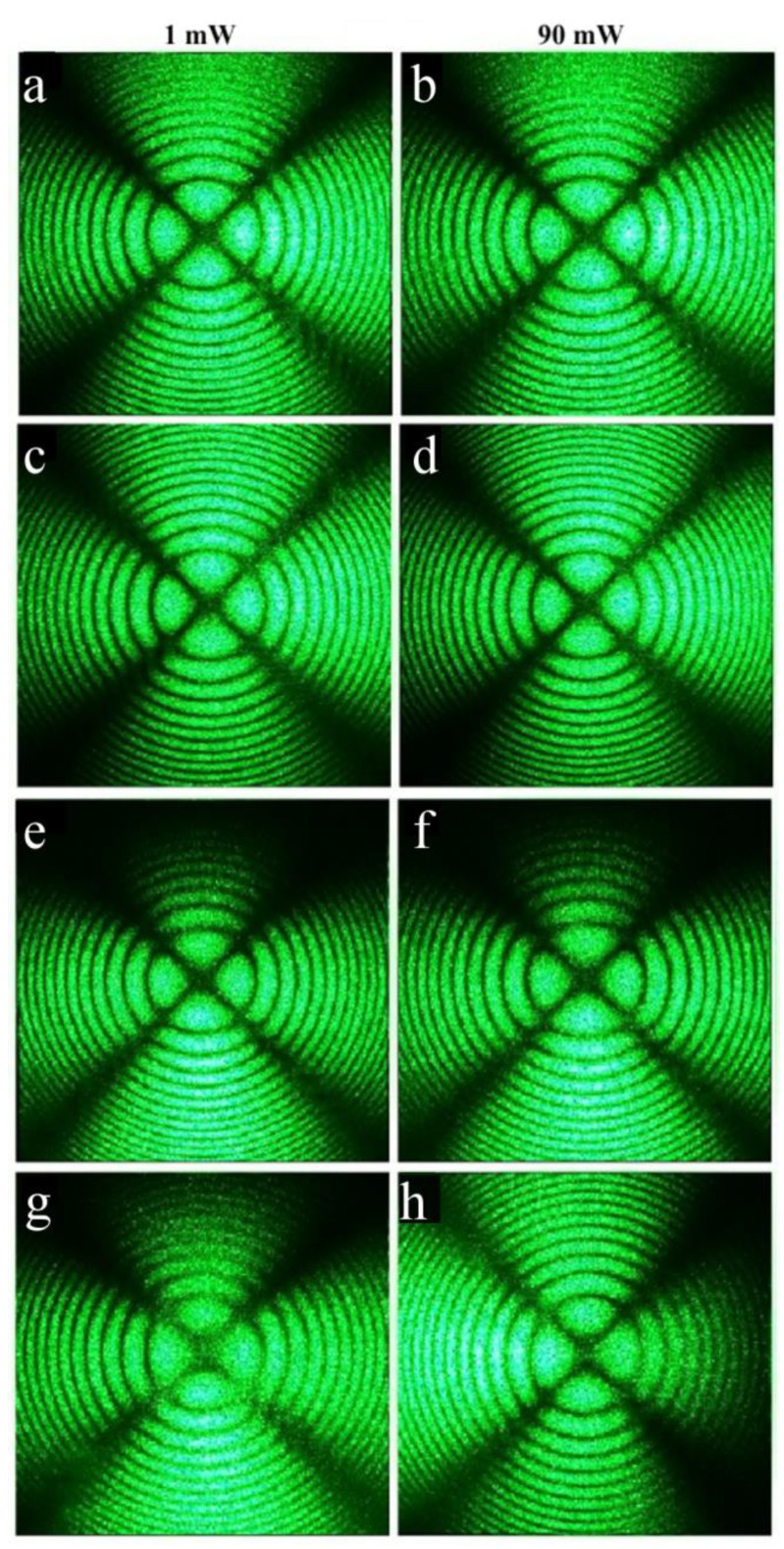
Conoscopic patterns of HG-doped LiNbO_3_:Mg,B samples: [Mg] = 4.2 (**a**,**b**), 3.9 (**c**,**d**), 3.7 (**e**,**f**), and 3.6 (**g**,**h**) mol%. Wafer thickness ~3 mm. λ = 532 nm. P = 1 and 90 mW.

As expected, the conoscopic patterns of the HG-doped LiNbO_3_:B:Mg crystals are less perfect than the patterns of the SP-doped LiNbO_3_:B:Mg crystals, Figure 14 and Figure 15. Branches of the Maltese cross have defects and signs of anomalous optical biaxiality, Figure 15a,b,e–h.

The patterns of the Mg,B3-HG and Mg,B4-HG crystals have the following defects: the Maltese cross is elongated in the vertical direction, corresponding to the direction of the deformation of the optical indicatrix of the crystal without a break in the center of the cross, and the isochromes take the form of ellipses, Figure 15e–h. There is a slight speckle structure in the pattern of the Mg,B1-HG crystal. The structure is especially noticeable in the region of the lower half-plane; it disappears as the laser radiation power increases to 6.3 W/cm^2^, Figure 15a,b. The conoscopic pattern of the Mg,B1-HG crystal improves slightly with an increase in the laser radiation power. Apparently, this is due to the healing of electronic defects by the laser beam. Defects on the branches of the Maltese cross, a deformation of the optical indicatrix of the crystal, and changes in the shapes of the isochromes are apparently due to the more developed microstructure of the HG-doped LiNbO_3_:B:Mg crystals, the inhomogeneity of the incorporation of the dopant into the crystals’ structures and, as a consequence, mechanical stresses. The inhomogeneity of dopant incorporation into HG-doped LiNbO_3_:B:Mg crystals is caused by a noticeable difference in the K_D_ value from unity over the entire dopant concentration range studied, Table 2.

Noticeable additional anomalies are absent from the conoscopic patterns of the HG-doped LiNbO_3_:Mg,B crystals with an increase in the laser radiation power, Figure 15. This is due, also for SP-doped LiNbO_3_:B:Mg crystals, to the absence of a noticeable photorefractive response in these crystals, Figure 12 and Figure 13.

Optical microscopy revealed an HG-doped LiNbO_3_:Mg:B crystal with the most uniform microstructure: Mg,B2-HG with a magnesium concentration of [Mg] = 3.9 mol%. Conoscopic patterns clearly confirm this: Mg,B2-HG has the most perfect conoscopic pattern, Figure 15c,d. This pattern corresponds to the standard conoscopic patterns of uniaxial crystals without noticeable signs of anomalous optical biaxiality.

**Figure 16:**
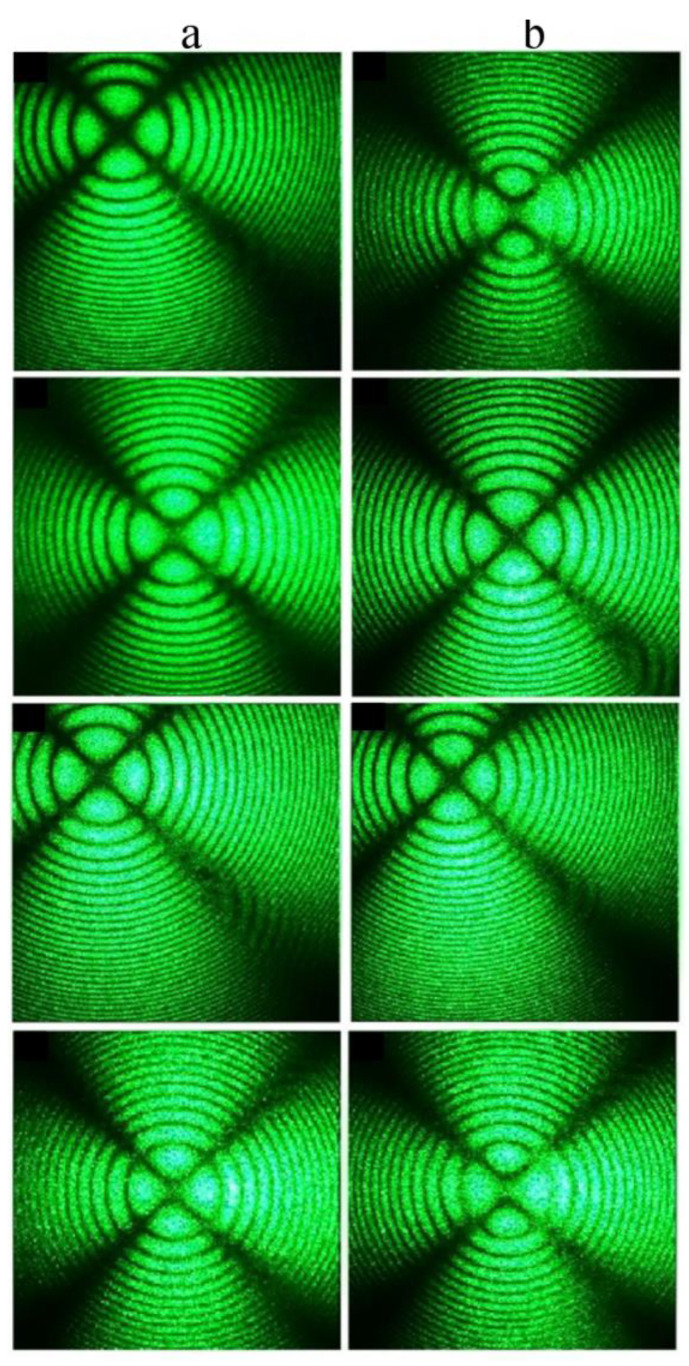
Conoscopic patterns of Mg,B4-HG crystal, [Mg] = 3.6 mol%, when scanning along the plane of the input face of a 3 mm wafer (**a**) and a parallelepiped that is 10 mm thick (**b**). λ = 532 nm. P = 90 mW.

The Mg,B4-HG crystal has the least uniform microstructure among the HG-doped LiNbO_3_:Mg:B crystals, Figure 9a. Conoscopic patterns agree with this: the Mg,B4-HG crystal indeed has the least perfect patterns, Figure 15g,h. Moreover, when scanning along the plane of the input face of this crystal sample, the conoscopic patterns differ markedly from each other, Figure 16. This indicates the presence of a significant optical non-uniformity of the studied crystal over the volume.

There are defects on the conoscopic patterns, and speckle structures are present on the branches of the Maltese cross, Figure 16. This indicates the non-uniformity of the incorporation of the dopant into the crystal structure. There are signs of anomalous optical biaxiality: the Maltese cross is elongated in the vertical direction, corresponding to the direction of deformation of the optical indicatrix of the crystal, there are translucences and even breaks in the center of the Maltese cross, and isochromes take the form of ellipses, Figure 16.

Our work clearly shows a good agreement between the results of the experimental studies obtained through various methods: physicochemical studies, full-profile XRD analysis, optical microscopy, optical spectroscopy, PILS, and laser conoscopy.

## 4. Conclusions

Two series of LiNbO_3_:Mg:B crystals were grown. The crystals were doped using the solid-phase (SP) and homogeneous (HG) methods. The dopant components were a non-photorefractive metallic dopant (Mg) and a non-metallic dopant (B). Mg reduces the photorefractive effect, and B also reduces the photorefractive sensitivity and increases the optical and compositional uniformity of the LiNbO_3_ crystal. LiNbO_3_:Mg crystals were grown using two methods of doping with a single non-photorefractive metal dopant, Mg. A control NSLN crystal was grown from a melt of a congruent composition (Li/Nb ≈ 0.946) with the addition of 5.5 wt% K_2_O to the melt via the HTTSSG method.

The distribution coefficient of magnesium, K_D_, in the HG-doped LiNbO_3_:Mg,B crystals is ~1.25–1.47 times higher than for the LiNbO_3_:Mg,B SP-doped crystals. Physicochemical and structural reasons were established for the difference in the K_D_ during the growth of the LiNbO_3_:B:Mg and LiNbO_3_:Mg crystals from SP- and HG-doped charges. It was shown that both the doping method and the presence of boron in the melt have a crucial effect on the physicochemical conditions of crystallization of Mg-containing LN crystals. The presence of boron in the melt, at least in the case of SP doping, significantly increases the compositional and optical uniformity of LiNbO_3_:B:Mg crystals since in the presence of boron that the K_D_ of magnesium approaches unity and is almost independent on the dopant’s concentration in the melt. Traces of boron generally have a positive effect on SP-doped LiNbO_3_:Mg,B crystals and a negative effect on HG-doped LiNbO_3_:Mg,B crystals.

A full-profile XRD analysis investigated the features of the defect structures and deformation of oxygen octahedra in LiNbO_3_:B:Mg and LiNbO_3_:Mg crystals obtained using different doping methods. Significant differences in the defect structures of LiNbO_3_:B:Mg and LiNbO_3_:Mg crystals of different geneses were unambiguously confirmed. In SP-doped LiNbO_3_:B:Mg and LiNbO_3_:Mg crystals, magnesium cations occupy only lithium sites, forming Mg_Li_ defects. At the same time, in HG-doped LiNbO_3_:B:Mg and LiNbO_3_:Mg crystals, magnesium cations occupy both lithium and vacant octahedra, forming Mg_Li_ and Mg_V_ defects.

The influence of the technology on the practically important characteristics of LiNbO_3_:B:Mg crystals was analyzed when obtaining a series of crystals with different dopant concentrations in a single technological cycle. It is shown that an additional complication of the melt structure arises and sequentially accumulates in the case of using the dilution method during the growth of a series of LiNbO_3_:B:Mg crystals. When diluted, the next crystal with a lower dopant concentration is obtained when a nominally pure lithium niobate charge of a congruent composition is added to the melt. The most defective sample among the studied LiNbO_3_:B:Mg crystals was the B:Mg4-HG crystal, with the lowest dopant concentration in the series of HG-doped crystals of ~3.6 mol%. The reason for this was the use of dilution technology in growing a series of LiNbO_3_:B:Mg crystals. The B:Mg4-HG crystal was the last in a series of HG-doped crystals; the melt during its growth contained the maximum number of types of ionic complexes. The complexes accumulated over the entire period of growth of a series of LiNbO_3_:B:Mg crystals. This led to the most non-equilibrium crystallization and, accordingly, an increase in the defectiveness of the crystal.

The effect of boron on the microstructures of LiNbO_3_:B:Mg crystals was studied. The absence of a stripe structure on Y-cuts is a practically important positive consequence of the presence of a strong complexing agent, boron, in the melt during the growth of SP- and HG-doped LiNbO_3_:Mg,B crystals. The presence of a stripe structure in the direction perpendicular to the growth axis of the LiNbO_3_:Mg crystals significantly reduces the potential for their commercial use as a material for laser radiation conversion devices.

When both SP and HG doping methods are used, a LiNbO_3_:Mg:B crystal with a magnesium concentration in the crystal close to [Mg] ≈ 3.9 mol% has the most uniform microstructure.

A study of the transmission spectra showed that for SP- and HG-doped LiNbO_3_:Mg,B crystals, the absorption edge shifts significantly to the short-wavelength region with an increase in the magnesium concentration. The absorption edge of the SP-doped LiNbO_3_:Mg,B crystals is slightly (~3.5 nm) shifted to short wavelengths when compared to the HG-doped LiNbO_3_:Mg,B crystals. This indicates a somewhat smaller number of charged electronic structural defects in the SP-doped LiNbO_3_:Mg,B crystals compared to the HG-doped LiNbO_3_:Mg,B crystals. This result in general correlates with the data on the study of the defect structures of crystals via the Rietveld method.

A PILS study of LiNbO_3_:Mg,B crystals showed that the photorefractive effect is already suppressed at a magnesium concentration of [MgO] = 2.56 mol% for SP-doped crystals and at [MgO] = 3.6 mol% for HG-doped crystals. The concentration threshold after which the photorefraction effect is strongly suppressed in LiNbO_3_:Mg crystals is near [Mg] ~ 5.5 mol% MgO. Thus, the traces of boron in LiNbO_3_:Mg,B crystals also affect the fact that the photorefractive sensitivity is suppressed at a much lower concentration of metallic magnesium dopant than in LiNbO_3_:Mg crystals.

Studies carried out via laser conoscopy confirm the rather high structural and compositional uniformities and, as a consequence, high optical uniformity of SP-doped LiNbO_3_:Mg,B crystals. The conoscopic patterns of all SP-doped LiNbO_3_:Mg,B samples correspond to the standard conoscopic patterns of uniaxial crystals. The conoscopic patterns of HG-doped LiNbO_3_:Mg,B crystals are less perfect than those of SP-doped LiNbO_3_:Mg,B crystals. There are defects on the branches of the Maltese cross, indicating an anomalous optical biaxiality. Thus, for Mg,B3-HG and Mg,B4-HG crystals, the Maltese cross is elongated in the vertical direction, corresponding to the direction of deformation of the optical indicatrix of the crystal, without a break in the center of the cross, and the isochromes take the form of ellipses. For the Mg,B1-HG crystal, an insignificant speckle structure is observed which is especially noticeable in the region of the lower half-plane of the conoscopic pattern.

For all studied HG-doped LiNbO_3_:B:Mg crystals, there are no noticeable additional anomalies in the conoscopic patterns with increasing laser radiation power. This is due to the absence of a noticeable photorefractive response in these crystals, as well as for the SP-doped LiNbO_3_:Mg,B crystals. Of all the studied HG-doped LiNbO_3_:B:Mg crystals, the Mg,B2-HG crystal has the most perfect conoscopic pattern without noticeable signs of anomalous optical biaxiality. Its pattern corresponds to the standard conoscopic patterns of uniaxial crystals. At the same time, the Mg,B2-HG crystal has the most uniform microstructure among the investigated HG-doped LiNbO_3_:B:Mg crystals. The least uniform microstructure is observed in the Mg,B4-HG crystal, and this crystal has the least perfect conoscopic pattern. Moreover, when scanning along the plane of the entrance face of the Mg,B4-HG crystal sample, conoscopic patterns were obtained that differ markedly from each other. This indicates the presence of a significant optical non-uniformity in the studied crystal over the volume.

Thus, the work determined the optimal technological approaches to growing optically uniform LiNbO_3_:B:Mg crystals with a high optical damage resistance. At the same time, a good agreement is unambiguously shown between the results of experimental studies obtained via various methods: physicochemical studies, full-profile XRD analysis, optical microscopy, optical spectroscopy, PILS, and laser conoscopy.

## Figures and Tables

**Table 1:** Impurity concentrations in the Nb_2_O_5_B:Mg precursor, LiNbO_3_:B:Mg charge, and LiNbO_3_:Mg charge obtained by HG and SP doping.

Elements		Mn	Ni	Al	Fe	Cr, Cu, V	Pb, Sn	Bi	Si, Ti, Mo, Ca, Co	Sb	Zr
C·10^−3^, wt%	Nb_2_O_5_:B:Mg precursor	<0.2	<0.2	<0.3	<0.3	0.25	<0.4	0.5	0.8	1.1	0.5
	HG charge LiNbO_3_:B:Mg	<0.3	<0.25	<0.5	<0.35	0.3	<0.5	0.5	0.9	1.4	1
	SP charge LiNbO_3_:B:Mg	<0.3	<0.3	<0.6	<0.4	0.3	<0.4	0.5	0.8	1.3	1
	SP charge LiNbO_3_:Mg	<0.2	<0.3	<0.4	<0.3	<0.35	<0.5	0.4	0.7	1.0	0.8
	HG charge LiNbO_3_:Mg	<0.2	<0.4	<0.5	<0.3	<0.2	<0.4	<0.5	0.6	0.9	0.7

**Table 2:** Impurity concentrations and distribution coefficients (K_D_) of magnesium in LiNbO_3_:B:Mg and LiNbO_3_:Mg crystals of various geneses and compositions.

	LiNbO_3_:B:Mg, mol%, HG	K_D_	LiNbO_3_:B:Mg, mol%, SP	K_D_	LiNbO_3_:Mg, mol%, SP	K_D_	LiNbO_3_:Mg, mol%, HG	K_D_
1	4.2	1.22	3.87	0.97	5.2	0.89	5.0	1.15
2	3.9	1.35	3.25	0.965	4.9	0.96	4.74	1.20
3	3.7	1.38	2.73	0.96	4.2	1.05		
4	3.6	1.47	2.56	0.96	3.4	1.15		

**Table 3:** Full-profile analysis results. Models for the locations of intrinsic and dopant defects in HG-doped LiNbO_3_:Mg:B crystals. Unit cell periods—*a, c*; magnesium concentration—C(Mg); weight profile—R_wp_ and profile R_p_-factor.

HG Doping									
C(Mg) = (2.5) 3.6 mol%; R_wp_(%) = 4.08, R_p_(%) = 5.21 *a* = 5.1469 Å, *c* = 13. 8578 Å					C(Mg) = 4.2 mol% R_wp_(%) = 8.16, R_p_(%) = 11.09 *a* = 5.1449 Å, *c* = 13. 8519 Å				
	G	*x/a*	*y/b*	*z/c*		G	*x/a*	*y/b*	*z/c*
Nb	0.96	0	0	0	Nb	0.934	0	0	0
O	1.0	0.04992	0.34165	0.0637	O	1.0	0.05595	0.3201	0.0661
Li	0.98	0	0	0.2811	Li	0.954	0	0	0.2779
Nb_Li_	0.01	0	0	0.2784	Nb_Li_	0.017	0	0	0.2832
Nb_V_	0.013	0	0	0.1210	Nb_V_	0.018	0	0	0.1495
Mg_Li_	0.01	0	0	0.2913	Mg_Li_	0.019	0	0	0.2700
Mg_V_	0.019	0	0	0.1469	Mg_V_	0.024	0	0	0.1590

**Table 4:** Full-profile analysis results. Models of the location of intrinsic and doping defects in SP-doped LiNbO_3_:Mg:B crystals. Unit cell periods—*a, c*; magnesium concentration—C(Mg); weight profile—R_wp_ and profile R_p_-factors.

SP Doping									
C_V_(Mg) = 3.25 mol%; R_wp_(%) = 4.68, R_p_(%) = 6.89 *a* = 5.1453 Å, *c* = 13. 8528 Å					C_V_(Mg) = 3.87 mol% R_wp_(%) = 3.83, R_p_(%) = 4.94 *a* = 5.1475 Å, *c* = 13. 8584 Å				
	G	*x/a*	*y/b*	*z/c*		G	*x/a*	*y/b*	*z/c*
Nb	0.96	0	0	0	Nb	0.94	0	0	0
O	1.0	0.0549	0.3361	0.0633	O	1.0	0.0519	0.3451	0.0643
Li	0.955	0	0	0.2810	Li	0.95	0	0	0.2812
Nb_Li_	0.01	0	0	0.2808	Nb_Li_	0.01	0	0	0.2759
Nb_V_	0.02	0	0	0.1040	Nb_V_	0.004	0	0	0.1140
Mg_Li_	0.035	0	0	0.2822	Mg_Li_	0.039	0	0	0.2800

**Table 5:** Interionic distances calculated for the studied LiNbO_3_:Mg:B crystals of different geneses and NSLN crystal.

Atom Pairs	NSLN	HG Doping		SP Doping	
C(Mg), mol%		3.6	4.2	3.25	3.87
Li-O, Nb-O Bond Length in LiO_6_, NbO_6_ octahedra					
Li-O	2.244	2.250	2.346	2.253	2.246
Li-O	2.143	2.075	2.131	2.112	2.074
Nb-O	2.099	2.126	2.148	2.135	2.110
Nb-O	1.839	1.866	1.777	1.831	1.882
Bond Length Nb_Li_-O, Mg_Li_-O					
Nb_Li_-O	2.270	2.277	2.294	2.255	2.299
Nb_Li_-O	2.132	2.062	2.154	2.111	2.050
Mg_Li_-O	-	2.151	2.426	2.241	2.256
Mg_Li_-O	-	2.127	2.102	2.118	2.068

**Table 6:** Values of the unit cell periods of LiNbO_3_:Mg crystals refined via full-profile analysis (Rietveld method).

Sample Number		Mg1-HG	Mg2-SP	Mg2-HG
LiNbO_3_:Mg	C, mol%	5.0	4.9	4.74
	*a*, Å	5.1503	5.1506	5.1488
	*c*, Å	13.8687	13.8693	13.8644

**Table 7:** Refined values of atomic coordinates (*x/a*, *y/b*, *z/c*) and site population factors G in LiNbO_3_:Mg crystals.

	G	*x/a*	*y/b*	*z/c*		G	*x/a*	*y/b*	*z/c*
Sample Mg1-HG: C = 5.0 mol%					Sample Mg2-SP: C = 4.9 mol%				
R_wp_(%) = 13.42; R_p_(%) = 11.18					R_wp_(%) = 10.57; R_p_(%) = 7.54				
Nb	0.91	0	0	0	Nb	0.91	0	0	0
O	1.00	0.0798	0.3308	0.0664	O	1.00	0.0726	0.3285	0.0649
Li	0.96	0	0	0.2836	Li	0.926	0	0	0.2852
Nb_Li_	0.013	0	0	0.2864	Nb_Li_	0.023	0	0	0.28
Nb_V_	0.025	0	0	0.13	Nb_V_	0.015	0	0	0.16
Mg_Li_	0.025	0	0	0.2712	Mg_Li_	0.06			0.123
Mg_V_	0.031	0	0	0.128	Mg_Vt_	0	0	0	
Sample Mg2-HG: C = 4.74 mol%									
R_wp_(%) = 11.44; R_p_(%) = 8.38									
Nb		0.91		0		0		0	
O		1		0.0642		0.3332		0.0664	
Li		0.95		0		0		0.292	
Nb_Li_		0.011		0		0		0.278	
Nb_V_		0.027		0		0		0.122	
Mg_Li_		0.025		0		0		0.27	
Mg_V_		0.02		0		0		0.127	

## Data Availability

The raw data required to reproduce these findings are available from corresponding author, M.P., upon reasonable request.
